# Giant Mesenteric Cyst and Right Sided Syndrome in a 15-Year-Old Boy

**DOI:** 10.1055/s-0039-1683993

**Published:** 2019-05-23

**Authors:** Ayanaw Tamene, Melkam Desta, Habeneyom Tebeje, Yeshiambel Getie, Hailemariam Berhane

**Affiliations:** 1 Department of Pediatrics and Child Health, Bahir Dar University, Bahir Dar, Bahir Dar, Ethiopia; 2 Department of Surgery, Bahir Dar University, Bahir Dar, Bahir Dar, Amhara, Ethiopia; 3 Department of Radiology, Bahir Dar University, Bahir Dar, Bahir Dar, Amhara, Ethiopia

**Keywords:** cryptorchidism, giant mesenteric cyst, renal agenesis, right side syndrome

## Abstract

Giant mesenteric cyst is a rare benign abdominal tumor. It usually arises from the mesenteric side of the small bowel. Right side syndrome is the term used for congenital absence of right kidney and right testis. We report on a 15-year-old male who presented with progressive abdominal distension, early satiety, and difficulty walking or running. Abdominal ultrasound and computed tomography (CT) revealed a giant mesenteric cyst, absence of the right kidney, and left moderate hydronephrosis. After excision of the cyst, the patient was fully recovered. Our report shows that both conditions may occur in the same patient and therefore an association of these two diseases cannot be excluded.

## Introduction

Mesenteric cysts are rare intra-abdominal benign tumors with no classical clinical feature. 1 They were first reported in 1507 by the Italian anatomist Benevieni after an autopsy of an 8 years old girl. 2 The frequency is 1 in 100, 000 to 350, 000 adult hospital admissions 3 4 and 1 in 20,000 to 35,000 pediatrics hospital admissions. 4 It mostly arises from the mesenteric border of small intestine. The size varies from 8 to 35 cm. 5 Commonly patients complain of abdominal swelling, abdominal pain, early satiety, vomiting, diarrhea, and even present with an acute abdomen. Abdominal computed tomography (CT) scan, magnetic resonance imaging (MRI) and abdominal ultrasound are diagnostic. Surgical complete excision of the cyst is the treatment of choice with a very low recurrence rate.

Right sided syndrome is a congenital absence of right kidney and right testis occurring in a single patient. Unilateral renal agenesis may be associated with anomalies of the genitourinary system, such as absence of testis, epididymis, and uterine anomalies. Again, abdominal ultrasound and MRI are diagnostic. 6

## Case Presentation

A 15 years old male presented with the compliant of progressive abdominal swelling since 10 years which had further progressed within the preceding 12 months.” Furthermore he experienced early satiety, unable to run, difficulty walking, shortness of breath in lying position, and significant subjective weight loss in 3 months duration. He was a grade VII student but discontinued school due to walking difficulty for the last 3 months. Two years prior to the current presentation he was admitted for with chylous ascites diagnosed by abdominal ultrasound at our hospital; however, a CT or MRI was not done at this time and a planned treatment with octreotide could not be performed as the patient left the hospital prior to that.

**Fig. 1:**
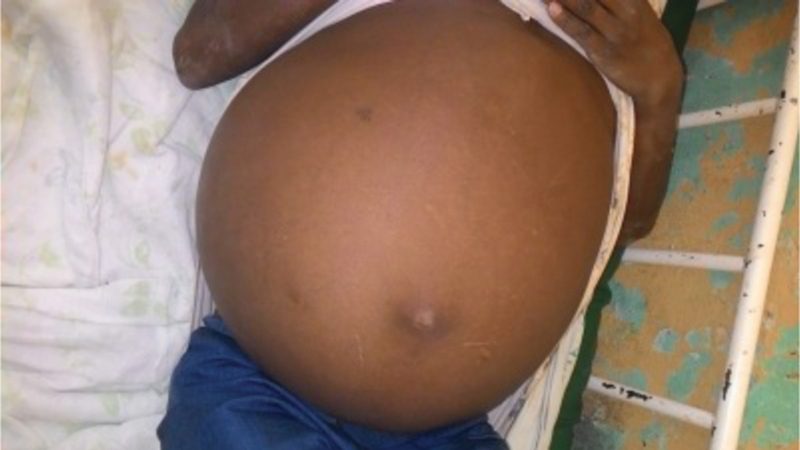
Significantly distended abdomen at presentation.

**Fig. 2:**
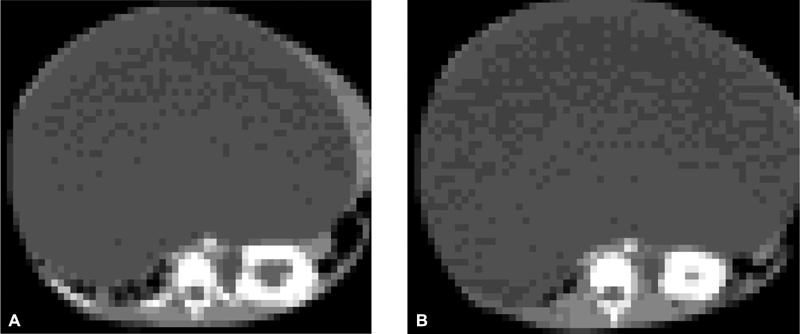
( **A** and **B** ) Axial post contrast CT scan of the abdomen, 20 × 32 cm huge cystic mass displacing the entire visceral organt to the back, absent right kidney and dilatation of renal pelvis and calyces of left kidney due to mass effect. CT, computed tomography.

On physical examination he was looking chronically sick, the vital signs were within normal limits. The abdomen was significantly distended ( Fig. 1 ) with dullness on percussion over the whole abdomen with a positive fluid thrill. The right scrotum was empty. The complete blood cell count, renal and liver function tests, and serum albumin and stool examination were normal. Abdominal ultrasound and abdominal CT scan showed a 20 × 32 cm giant mesenteric cyst, absent right kidney and left moderate hydronephrosis due to the compression effect from the mass ( Fig. 2 ). On exploratory laparotomy through a midline abdominal incision a huge retroperitoneal cyst from duodenum to sacrum was found. The cyst was completely excised and right orchiectomy was done for intra-abdominal testis to prevent testicular germ cell malignancy. Gram stain, culture, and gene expert of the cystic fluid was negative with 7/mm ^3^ white blood cells. A postoperative abdominal ultrasound postoperative day 7 showed a mild left sided hydronephrosis. The patient recovered uneventfully and was discharged 1 day later. Histology of the resected testis showed an atrophic testis without sign of malignant transformation. Follow-up on 20th post surgery was unremarkable.

## Discussion

Mesenteric cysts can arise from jejunum to the rectum, mostly from mesenteric border of the ileal mesentery. The possible etiology includes a benign proliferation of ectopic mesenteric lymphatic vessels which lack communication with the remaining lymphatic system, 7 trauma, surgery, and neoplasms. 8 It can occur at any age and approximately one-third of cases are found in children younger than 15 years. 9 Mesenteric cysts in pediatrics age group are seen often in males (62.5%). The majority of the patients are younger than 10 years, and 75% younger than 5 years of age. 7 The clinical presentation is nonspecific and depends on the size and the site of the cyst.

Commonly, patients complain of abdominal swelling with or without pain, early satiety, vomiting, diarrhea, dyspepsia, and constipation. In extreme cases, infection or rupture associated with an acute abdomen has been reported. 10 11 Our patient showed abdominal painful swelling, early satiety and walking difficulty. The 10-year duration of symptoms is explained by the slow growth of the cyst and diagnostic difficulty of mesenteric cysts by primary health care providers. Abdominal CT scan, abdominal ultrasound, and MRI are diagnostic for mesenteric cysts. 10 12

Surgical complete excision of the cyst is the treatment of choice 13 14 with very low recurrence rates and prevents malignant transformation into adenocarcinoma. 15 16 Simple drainage and marsupialization are also the treatment options with unacceptable recurrence and infection. 7

The term right sided syndrome is used when the absence of the right kidney and right testis occurs in a single patient. 17 The kidney and the testis are derived from the intermediate mesoderm during embryogenesis. Renal agenesis may be unilateral or bilateral occurring from failure of induction by the ureteric bud or errors in development of the mesonephric duct. 18 Unilateral renal agenesis is a developmental defect associated with anomalies of the genitourinary system, such as absence of testis (cryptorchidism), epididymis, and anal, vertebral, or uterine anomaly. 17 18 The gold standard for diagnosis of a solitary kidney after detection on ultrasound is MRI. 19 Individuals who have solitary kidney should be informed about the case and regular follow-up must be adjusted. All possible nephrotoxic drugs have to avoid as much as possible.

In our case, abdominal ultrasound was suggestive for huge mesenteric cyst and abdominal CT scan showed 20 × 32 cm giant mesenteric cyst, absent right kidney, and left moderate hydronephrosis due to compression effect from the cyst. Createnine and blood urea nitrogen were normal. The patient underwent open surgery through midline access. The cyst excised completely and right orchiectomy was done and the patient recovered uneventfully. The probability of a malignant neoplasm developing in an undescended testicle is approximately 20 to 48 times greater than a normally descended testicle and higher (approximately 5%) for intra-abdominal testicles. 20 21 Testicular tumor in intra-abdominal testis can occur as early as 1st year of life and higher frequency starting from puberty. 22 In cases of testicular atrophy, it must be considered whether orchiectomy, a testicular prosthesis or a close follow-up regime for detecting carcinoma-in-situ-testis or invasive testicular neoplasia are options of treatment. 23 24 In our patient the testis was atrophied and orchiectomy was done as there is no prosthesis and limitation of close follow-up and early detection of neoplasms. 25 The left hydronephrosis resolved postoperatively evidenced on subsequent ultrasounds. To the best of our knowledge, there is no case report of giant mesenteric cyst and right sided syndrome in a single patient.

## Conclusion

In conclusion, we present the first coincidental finding of a right sided syndrome and giant mesenteric cyst. Whether this observation represents a true association between two diseases remains unknown. However, in children with renal agenesis and ipsilateral cryptorchidism surgeons may also expect additional abdominal pathology, such as mesenteric cysts.

## Notes

New Insights and the Importance for the Pediatric Surgeon Giant mesenteric cyst can be symptomatic for prolonged duration more than 12 months (for10 years) without life threatening complication. Right sided syndrome and giant mesenteric cyst can occur in a single patient with unknown association which need for explanation.
